# Antigen-Specific B Memory Cell Responses to *Plasmodium falciparum* Malaria Antigens and *Schistosoma haematobium* Antigens in Co-Infected Malian Children

**DOI:** 10.1371/journal.pone.0037868

**Published:** 2012-06-05

**Authors:** Kirsten E. Lyke, Amy Wang, Abdoulaye Dabo, Charles Arama, Modibo Daou, Issa Diarra, Christopher V. Plowe, Ogobara K. Doumbo, Marcelo B. Sztein

**Affiliations:** 1 Center for Vaccine Development, University of Maryland School of Medicine, Baltimore, Maryland, United States of America; 2 Malaria Research and Training Center, University of Bamako, Bamako, Mali; 3 Department of Immunology, University of Stockholm, Stockholm, Sweden; 4 Howard Hughes Medical Institute, Baltimore, Maryland, United States of America; University of Massachusetts Medical School, United States of America

## Abstract

Polyparasitism is common in the developing world. We have previously demonstrated that schistosomiasis-positive (SP) Malian children have age-dependent protection from malaria compared to matched schistosomiasis-negative (SN) children. Evidence of durable immunologic memory to malaria antigens is conflicting, particularly in young children and the effect of concomitant schistomiasis upon acquisition of memory is unknown. We examined antigen-specific B memory cell (MBC) frequencies (expressed as percentage of total number of IgG-secreting cells) in 84 Malian children aged 4–14 to malaria blood-stage antigens, apical membrane antigen 1 (AMA-1) and merozoite surface protein 1 (MSP-1) and to schistosomal antigens, Soluble Worm Antigenic Preparation (SWAP) and Schistosoma Egg Antigen (SEA), at a time point during the malaria transmission season and a follow-up dry season visit. We demonstrate, for the first time, MBC responses to *S. haematobium* antigens in Malian children with urinary egg excretion and provide evidence of seasonal acquisition of immunologic memory, age-associated differences in MBC acquisition, and correlation with circulating *S. haematobium* antibody. Moreover, the presence of a parasitic co-infection resulted in older children, aged 9–14 years, with underlying *S. haematobium* infection having significantly more MBC response to malaria antigens (AMA1 and MSP1) than their age-matched SN counterparts. We conclude that detectable MBC response can be measured against both malaria and schistosomal antigens and that the presence of *S. haematobium* may be associated with enhanced MBC induction in an age-specific manner.

## Introduction

The acquisition of immunologic memory depends upon a rapid and specific recall response after re-exposure to an antigen. A component of immunological memory that is central to long-term humoral immunity is the memory B cell response (MBC). MBC differentiate into antibody-secreting plasma cells that enable long-term maintenance of serum antibody levels. Long-lived plasma cells (LLPC) may reside in sequestered niches with limited space such as the bone marrow. Newly-generated plasmablasts would periodically displace these LLPC resulting in a slow decline of the compartment over time (i.e. the plasma cell niche competition model) or programming of individual plasma cells at the time of induction may determine differential lifespan (the plasma cell imprinted lifespan model) (Reviewed by Slifka) [1]. The sustained generation of antibody depends upon either intermittent or persistent antigen exposure, as seen in repeated or chronic infections, leading to MBC differentiation into effector plasma cells, or polyclonal antigen-independent differentiation of MBC via Toll-like receptor (TLR) or T-cell dependent bystander activation [2]. Very little is known about the acquisition of immunologic MBC to parasites. Knowledge gained regarding the acquisition of memory to parasites, are of great importance for the development of novel vaccines to both malaria and helminthes.

Evidence of durable immunologic memory to malaria antigens is mixed, particularly in young children, where repetitive and ongoing exposure to malaria is required to achieve and maintain immunity [3], [4]. Maternally-derived antibody to *P. falciparum* protects infants in the early months of life [5], [6] after which, acquisition of antibody is critical to naturally-acquired blood-stage malaria immunity [7], [8]. It has been assumed that because antibodies are short-lived and decrease rapidly after infection, long-term immunologic memory acquisition may be inefficient. MBC to blood-stage malaria antigens were measured in a highly malaria-endemic area but at a reduced level compared to a more potent antigen-stimuli, (e.g., tetanus toxoid [9]). However, recent studies demonstrated long-lived MBC responses to *P. falciparum (Pf)* and *P. vivax (Pv)* antigens in an area of low-endemicity [10], as well as an expanding *Pf* MBC compartment elicited by repeated malaria exposure [11].

In chronic human *Shistosoma spp.* infections, antibody and age correlate with resistance to re-infection [12]–[14]. *S. haematobium* prevalence has risen sharply in West Africa, due in large part to the building of hydroelectric dams [15]. Chronic egg-laying schistosomes exert a persistent stimulatory effect on the host immune system, chiefly to egg antigens. *S. mansoni* egg production correlates with increased levels of the C3d component of complement (B cell receptor, CD21 ligand) [16], and enhances TLR9 responsiveness of bone marrow-derived Th2-primed macrophages [17], both of which have been shown to be potent inducers of MBC [18], [19]. However, to our knowledge, MBC to *Schistosoma spp.* have never been measured in an endemic population.

We and others have shown that *S. haematobium* infection to protects against uncomplicated *Pf* malaria in an age-specific manner in West African children [20], [21]. Both parasitic diseases elicit stage-specific immunological responses in the human host [22]–[24]; however, dual infection may disrupt immunologic homeostasis and either enhance or abrogate pathogenicity [25]–[28]. We report here on the appearance and persistence of MBC over the course of a single malaria transmission season and measure MBC to both blood-stage malaria antigens and, for the first time, *S. haematobium* antigens. Given that one parasitic infection may immunologically perturb the immune responsiveness to another infection in the human host, we also examined the effect of concomitant schistosomiasis upon the acquisition of memory to malaria.

## Methods

### Specimens and Surveillance

Bandiagara (pop. ∼13,600) is located in Mali, West Africa and has intense seasonal transmission (July-December) of *P. falciparum* malaria and *S. haematobium*. The entomologic inoculation rate is 20–60 infected bites per month during peak transmission, falling to near zero during the dry season. Children experience a mean of 1.54 symptomatic malaria episodes per year [29]. *S. haematobium* prevalence is 50% by the age of 14 years [20] with peak acquisition between the ages of 6–14 years [15]. The study was conducted over two sequential malaria seasons in 2002–2003 (half the matched children were enrolled in each year). No difference in rainfall pattern or malaria illness was noted between the two years. Study details have previously been reported [20], [25]. Briefly, baseline demographic data, urine samples and stool samples were collected in all children at screening. Morning urine samples were obtained and 10 milliliters filtered through Nucleopore® (Acton, MA) filters with Whatmann® filter paper (22 µm). Filter papers were stained with 5% Ninhydrin and examined for the presence of *S. haematobium* eggs when dry. Each individual submitted 3 sequential morning urine samples to improve sensitivity. Stool samples were collected and processed using the Kato-Katz method for parasite detection. Slides were read and intestinal parasites quantified. All children displaying symptomatic effects related to underlying *S. haematobium* infection (gross hematuria, renal insufficiency, peripheral edema) were offered praziquantel® treatment and excluded (n = 6). Exclusion criteria were presence of *S. mansoni* eggs in stool, evidence of acute or chronic illness, participation in prior malaria vaccine studies and pregnancy. Previous studies performed in Bandiagaran children from revealed no detectable filarial infections (i.e., *Loa loa, Onchocerca volvulus, or Wuchereria bancrofti)*. HIV prevalence is less than 2% among adults in Mali and this is found primarily in commercial sex workers. Study children were not tested individually for these infections although every attempt was made to only enroll healthy individuals. Children aged 4–14 years of age, diagnosed as having asymptomatic *S. haematobium* infection (SP), were matched by age, gender and residence to a child without schistosomiasis (SN) prior to the start of malaria transmission. Children were followed weekly, at a scheduled follow-up visit, over the malaria transmission season (25 weeks) and at a dry season follow-up appointment (∼9 months after enrollment at a time when standing water pools had dried and schistosoma transmission had ceased). The primary endpoint of the clinical study was time to first clinical malaria episode. A clinical episode of malaria was defined as *P. falciparum* parasitemia and axillary temperature ≥37.5°C on active surveillance, or parasitemia and symptoms leading to treatment-seeking behavior in the absence of other clear cause on passive surveillance. Clinic personnel were available 24 hours-a-day throughout study duration to detect, examine and treat malaria episodes. All children were pre-treated with albendazole to eliminate concomitant helminth infections and study samples were drawn at the time of their first clinical malaria episode (or at study week 25/Day 175 in the absence of a clinical infection) and again at the final dry season appointment. Monthly hemoglobin levels were checked to track evidence of anemia. Children were optimally treated for schistosomiasis with praziquantel at the final appointment (eight weeks after standing water pools dried, to ensure maturation of underlying schistosomula in individuals)

### Ethics

The trial was conducted in compliance with the Declaration of Helsinki. Study protocols were reviewed and approved by the University of Bamako’s Institutional Review Board (IRB) as well as the University of Maryland IRB. Village permission to conduct research was obtained from village chiefs, government officials and traditional healers prior to study initiation. Individual written informed consent was obtained from the parent or legal guardian of each child prior to screening and enrollment. *S. haematobium* is a chronic disease in the absence of therapy. Genito-urinary inflammation secondary to migrating eggs is usually sustained over decades although the majority of individuals never experience clinical morbidity. After consultation with experts and IRBs, a nine-month interval between detection and treatment of asymptomatic infections was felt to be safe and reasonable. Schistosomiasis transmission is seasonal in Mali. The efficiency of transmission rises in the dry season when standing water evaporates and cercariae are concentrated. Immature schistosomula are resistant to praziquantel and require eight weeks for larval maturation. Premature treatment of infections and treatment during ongoing transmission ensures a high degree of treatment failure and/or re-infection [30]. The consequences of treatment in endemic populations with ongoing, frequent parasitic exposure are unclear. Acquired partial resistance to infection occurs with down-regulation of inflammatory response to parasites [31], [32]. Treatment may interrupt this partial resistance and trigger a surge of immunologically mediated disease upon re-exposure. We proposed a measured approach ensuring the best clinical cure and least harm to our study subjects. Although a small risk of acute inflammation due to egg migration existed, all subjects were monitored for evidence of gross hematuria or symptoms of genitourinary pathology and were treated immediately with praziquantel (40 mg/kg) therapy in the event that symptoms developed.

### Sample Collection

Patient whole blood (5–10 mL) was collected at the study clinic into sterile Eppendorf and EDTA tubes on admission, prior to institution of anti-malarial therapy, and immediately refrigerated. Serum was processed as previously described [25]. Blood was processed by density centrifugation, within two hours of acquisition, utilizing lymphocyte separation medium (ICN Biomedical Inc, Aurora, OH) following standard techniques [33]. Peripheral blood mononuclear cells (PBMC) were resuspended in media and linear-rate frozen using isopropyl alcohol containers (Nalgene, USA) to–70°C overnight at the field site before next day placement in vapor-phase liquid nitrogen and transfer to the University of Maryland at Baltimore. Samples for this experiment were randomly chosen from the pool of samples that had at least 10.0×10^6^ cells as well as adequate quantity of sera for each child at a both the wet and dry season time points (n = 168). An investigator unblinded to the age of the study sample participants, randomly chose age-matched samples from the samples meeting defined criteria (n = 84). The wet and dry season time points were run simultaneously by blinded investigators.

### Antigen

Soluble egg antigen (SEA) and soluble worm antigen protein (SWAP) specific for *S. haematobium* were obtained through the Schistosome Related Reagent Repository (SR3) under the direction of Dr. Fred Lewis and sponsored by the National Institute of Allergy and Infectious Disease. Apical membrane antigen 1 (AMA1) [34] and merozoite surface protein 1 (42) were kindly donated by the Walter Reed Armed Institutes of Research (WRAIR). The malaria antigens were chosen because they were previously the foundation of several vaccine studies at the Malian field site.

### PBMC Expansion

PBMC expansion was performed as described previously [35], [36]. PBMC were thawed, washed and expanded for 5 days at 37°C, 5% CO_2_, in 6-well sterile plates (5×10^5^ cells/well) in a mitogen broth composed of 1/100,000 pokeweed mitogen (PWM) (Emory University), 50 µM β-mercaptoethanol (β-Me) (Sigma-Aldrich, St. Louis, MO), 6 µg/mL CpG-2006 (Qiagen/Operon, Huntsville, AL) and 1/10,000 *Staphylococcus aureus* Cowan (SAC) (Sigma-Aldrich, St. Louis, MO) in cRPMI in a total volume of 6 mL/well.

### Memory B Cell Assays

96-well ELISpot MAHA (Millipore, Billerica, MA) plates were coated in triplicate with AMA1 (5 µg/mL), MSP1_19_ (10 µg/mL), SEA (10 µg/mL), SWAP (10 µg/mL), tetanus toxoid (5 µg/mL) and total goat anti-human IgG (5 µg/mL) in phosphate buffered saline (PBS) overnight at 4°C, blocked with 1% bovine serum albumin (BSA) (Sigma) in RPMI for 2 h at room temperature (RT), washed with PBS, and incubated with 2×10^5^ expanded PBMC/well for wells coated with malaria or schistosoma antigens or tetanus toxoid. Dilutions for IgG wells were performed in sextuplet in serial two-fold dilutions starting at 10,000 cells/well and continuing to 1,250 cells/well using an optimized protocol. Cells were incubated for 6 h at 37°C, 5% CO_2_, washed with ddH_2_O followed by PBS+0.05% Tween 20 (PBST) and incubated with mouse anti-human pan-IgG-biotin conjugate antibody (Hybridoma Reagent Laboratory, Baltimore, MD) overnight at 4°C. Cells were washed with PBST, labeled with 1∶1000 horseradish peroxidase (HRP) conjugated Avidin D (Vector Laboratories, Burlingame, CA) for 1 h at RT. Cells were washed, stained with 3-Amino-9-ethylcarbazole (AEC) (Calbiochem, Billerica, MA) for 15 minutes at RT before the reaction was stopped with ddH_2_O. ELISpots were read using an automated reader (Immunospot®, Cellular Technology Ltd.). The final results are reported as the ratio of spot forming cells (SFC)/10^6^ expanded cells vs. total IgG SFC/10^6^ expanded cells henceforth termed antigen specific cell (ASC) ratio. Values >0.01 were considered positive.

### Elisa

#### Antibody Levels were Measured in Sera Using Optimized Standard Enzyme-Linked Immunosorbent Assay

(ELISA) assay on all clinical samples. Wells in Falcon Pro-Bind 96-well plates were coated with antigen (0.5 mg/mL AMA1, 1 mg/mL MSP1, 2 mg/mL SEA and 5 mg/mL SWAP) for 3 h, washed and blocked with PBS containing 0.05% Tween 20 and 10% nonfat dried milk. Sera samples, diluted to a concentration of 1∶10,000 for AMA1, 1∶5000 for MSP1, 1∶1000 for SEA and SWAP, were plated in duplicate, and developed with 1∶5000 HRP-conjugated goat anti-human IgG (KPL, Inc., Gaithersburg, MD) followed by TMB substrate solution (KPL, Inc., Gaithersburg, MD). If necessary, experiments were repeated with sera diluted a further 10-fold for accurate calculation of OD values. Phosphoric acid 1 M was used to stop the reaction and the optical density (OD) was read at 450 nm after 20 minutes. Background ODs from uncoated wells were subtracted from those of antigen-coated wells to adjust for nonspecific binding. Lower limits of detection were set at dilutions of 1∶500 for AMA1, 1∶250 for MSP1, and 1∶50 for SEA and SWAP. Sera from malaria-naive volunteers from the United States were used as negative controls and pooled sera from 10 local Malian adults (with known malaria episodes within 60 days prior to sera collection) were used as positive controls for both malaria and schistosoma antigens. The prevalence for *S. haematobium* is >50% in adults from Bandiagara and all individuals have seasonal exposure to malaria. A pool of sera derived from hyperimmune Malian adults served as a positive control. A pool of sera from unexposed U.S. adults served as a negative control as we could not be sure that any residents of Mali would be true negative controls. Sera differing in value by >20% in one or more of the replicate wells were retested.

### Flow Cytometry Staining and Analysis

Expanded and unexpanded PBMC were stained with fluorochrome-labeled monoclonal mouse anti-human antibodies against surface antigens (CD3-Energy Coupled Dye (ECD, Beckman Coulter, clone UCHT1), CD19-phycoerythrin (PE)-Cy5 (Beckman Coulter, clone TüK4), CD27-Alexa 700 (Beckman Coulter Custom Conjugate, Clone 1A4CD27), CD38-allophycocyanin (APC) (BD Biosciences, clone HIT2), and CD14-Pacific Blue (clone TüK4). Cells were washed and fixed in 1% paraformadehyde. Cells were analyzed using a MoFlo flow cytometer/cell sorter system (Beckman Coulter). List-mode data files were analyzed using WinList 7.0 3D (Verity Software House, Topsham, ME) software. An amine reactive dye (ViViD, Invitrogen, Oregon) was used as a dead cell discriminator and doublets/aggregates were subtracted from analysis. Gate placement was determined with the aid of Fluorescence Minus One (FMO) controls.

### Statistical Analysis

To ensure that the expansion of PBMC was adequate to allow for the detection of low numbers of antigen-specific MBC, we excluded specimens from analysis with <15,000 total IgG SFC/10^6^ expanded cells (arbitrarily defined as the bottom 10^th^ percentile of IgG SFC detected in experiments) [37]. Two samples were eliminated as a result of this criterion. The mean number of SFC from triplicate runs minus the number of spots from a control well (PBS) was used in the statistical analysis. The limit of detection (LOD) was defined as 1 antigen-specific SFC per well, which corresponded to 10 SFC per 10^6^ mononuclear cells when seeding 200,000 cells per well., Positive responses were defined as those that exceeded a threshold level of 0.01%; this cut off was defined by taking into the account the LOD (10 antigen-specific SFC/10^6^ PBMC) and the median total IgG SFC (51,000/106 PBMC) obtained during assay validation with study specimens (0.0098 rounded up to 0.01%) [37].

Statistical analysis was performed on GraphPad Prism 5 (Graphpad Software, Inc., San Diego, CA) and demographic and immunologic data were stratified and evaluated by season, schistosomiasis status and age stratification (age 4–8 and 9–14 years). Mann-Whitney rank sum analysis was used for continuous data not normally distributed and two-sided Student’s *t*-test for paired seasonal data. χ^2^ analysis, using Fisher’s exact test (two-tailed) as appropriate, was performed for categorical data. Spearman rank correlation coefficient was calculated utilizing GraphPad Prism 5. Multivariable Cox regression analysis controlling for age and schistosoma status was utilized for time to event analysis. A significance level of P≤0.05 was considered statistically significant. Adjustments were not made for multiple comparisons.

## Results

### Study Population

PBMC from 84 children aged 4–14 years (mean: 8.5 years) were examined. Demographic data of children determined to be *S. haematobium*-positive (SP) or *S. haematobium*-negative (SN) are depicted in Table 1. Among these, 54 were SP children (40 of whom developed malaria and 14 who did not acquire malaria during the transmission season) and 30 were SN children. Very few SN children remained malaria-free throughout the malaria transmission season and were not analyzed as a separate group. All children residing in Bandiagara are exposed to and likely acquire malaria many times over their lifetime [29], and no difference in antibody titer or MBC response was noted between SP children who had or had not developed malaria during the study period for any antigen in any age group (Table S1). Therefore, study results were combined into a single SP group. Samples were excluded if the viability or quantity of PBMC was insufficient or due to lack of detectable total IgG spots (N = 2). The mean PBMC viability after thaw was calculated as 86.3%. We previously reported on the finding of age-specific protection against the acquisition of malaria in SP children aged 4–8 years [20], so demographic and immunologic data were stratified and evaluated by age group in this study subset. No statistical differences were noted between age groups for SP children in the amount of eggs excreted per 10 ml aliquot of urine, the number of malaria episodes experienced or the time to first clinical malaria episode. SP children who developed malaria had a statistically longer time to first clinical malaria episodes (76 vs. 26 days, P<0.0001), and reduced total clinical malaria episodes (1.5 vs. 2.1, P = 0.002) as compared to age-matched SN children but similar levels of parasite density measured at the time of the first clinical malaria episode. There were no differences in the amount of eggs excreted between SP children who developed malaria and SP children who did not develop malaria in a single transmission season.

**Table 1:** Demographic characteristics at enrollment and features of *P. falciparum* clinical malaria episodes of *Schistosoma haematobium*-positive and age-matched *S. haematobium*-negative Malian children contributing PBMC for immunologic analysis.^a^

Category	Age (years)	*S. haematobium* (+)	*S. haematobium* (−)	P value
**Mean age (n)**	All ages	8.7 (54)	9.0 (30)	ns
	4–8	6.4 (27)	6.8 (15)	ns
	9–14	11.0 (27)	11.3 (15)	ns
**Female (%)**	4–14	26 (48.1)	16 (53.3)	ns
**Eggs (range)** a	4–14	58 (2–786)	0	n/a
	4–8	51 (2–596)	0	n/a
	9–14	66 (2–786)	0	n/a
**Clinical malaria episodes** b **(n, range)**	4–14	1.5 (1–4)	2.1 (1–4)	**0.002**
**Days to first clinical malaria episode** b **(range)**	4–14	76.2 (52–166)	26.4 (2–117)	**<0.0001** c
**Parasitemia** b,d **(range)**	4–14	9,299 (275–155,425)	11,785 (600–135,000)	0.87

a Urinary egg excretion detected in 10 ml of filtered morning urine.

b Results for children who did not develop malaria (n = 14) are not included these calculations. If no statistical difference was noted between children in the 4–8 year old category compared to the 9–14 year old category, the results were combined.

c Multivariable Cox regression analysis used controlling for age and schistosoma status.

d Geometric mean parasite density per mm^3^.

**Table 2:** Memory B cell (MBC) expressed as the mean number of malaria antigen specific SFC cells to apical membrane antigen (AMA1) or merozoite surface protein 1 (MSP1) compared to total IgG SFC (i.e., ASC ratio) measured in children with (SP) or without (SN) *S. haematobium* infection and stratified by age group and season (i.e., malaria transmission and dry season).

Malaria Antigen	Age (years)	Group^a^	MBC Transmission	MBC Dry	P value^b^
AMA1	4–14	SP + SN	**0.071**	**0.056**	0.16
		SP	**0.085**	**0.061**	0.08
		SN	**0.048**	**0.046**	0.94
	4–8	SP + SN	**0.063**	**0.036**	**0.05**
		SP	**0.070**	**0.045**	0.11
		SN	**0.049**	**0.020**	0.28
	9–14	SP + SN	**0.079**	**0.074**	0.77
		SP	**0.096** c	**0.074**	0.35
		SN	**0.050**	**0.073**	0.41
MSP1	4–14	SP + SN	**0.026**	**0.024**	0.69
		SP	**0.031**	**0.019**	**0.04**
		SN	**0.017**	**0.033**	0.15
	4–8	SP + SN	**0.019**	0.009	0.07
		SP	**0.022**	0.008	**0.049**
		SN	**0.013**	**0.011**	0.86
	9–14	SP + SN	**0.032**	**0.038**	0.53
		SP	**0.038**	**0.029**	0.12
		SN	**0.023**	**0.054**	0.12

a A total of 53 SP (26 aged 4–8 years, 27 aged 9–14 years) and 29 SN (14 aged 4–8 years, 15 aged 9–14 years) were examined in both the transmission and dry season (Note: Sample for 1 SP and 1 SN were excluded from the wet and from the dry season for a total of 4 samples).

b Statistical analysis performed between transmission and dry season values using paired ttest (two-tailed). P value deemed statistically significant ≤0.05.

c Statistical significance measured between SP and SN values using the Mann Whitney test for values not normally distributed (P value ≤0.05).

ASC ratios ≥0.01 were defined as a positive specific MBC responses. Mean age-stratified group values ≥0.01 are depicted in bold font.

**Figure 1:**
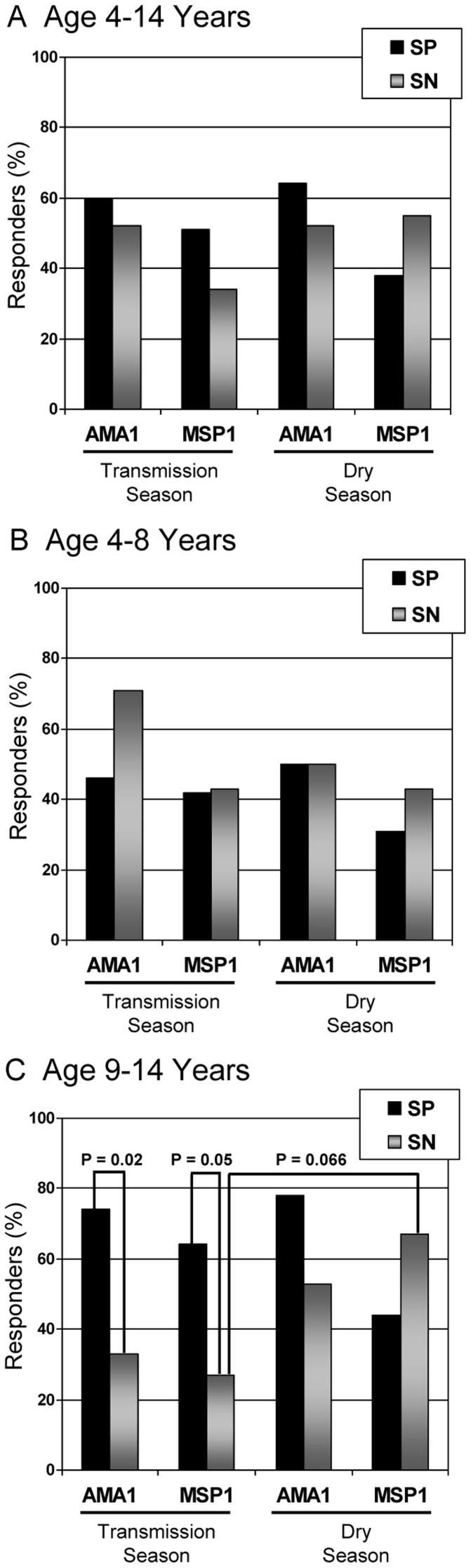
B memory cell responders to *P. falciparum* antigens. Shown are the percentages of B memory cell responders to *P. falciparum* antigens, AMA1 and MSP1, as determined by ELISpot assay during the malaria transmission season and again during the dry season with significant differences demarcated. Results were stratified by age (A–C) and *S. haematobium* status [*S. haematobium* positive (SP) = 54 and *S. haematobium* negative (SN) = 29] Responders are defined as those individuals with antigen specific cell (ASC) ratios (ratio of spot forming cells (SFC)/10^6^ expanded cells vs. total IgG SFC/10^6^ cells) greater than 0.01.

### Memory B cell Response to Malaria Antigens

Paired PBMC sample obtained at two time points (malaria transmission (wet) season and dry season) were examined in all children (Table 2, Figure 1A–C). The ASC ratio was calculated measuring detectable AMA1- and MSP1-specific MBC and expressed as either 1) a discrete value or 2) as a positive (defined as ≥0.01) response. The mean ASC ratio (Table 2) or total percentage of responders (Figure 1) stratified by time point, age group and *S. haematobium* status is depicted. Upon examination of all children, aged 4–14 years, regardless of schistosoma status, 47/82 (57.3%) had a detectable MBC to AMA1 and 36/82 (43.9%) to MSP1. There was no difference between SP children who had or had not acquired malaria, at any age group and in either season, during the study period (Table S1). Nine months later at the dry season follow-up, 49/82 (59.8%) had a detctable MBC to AMA1 and 36/82 (43.9%) to MSP1. When age groups were stratified by season, children aged 4–8 years did not have marked differences in MBC during the transmission season compared to children aged 9–14 years in either AMA1 [22/40 (55%) vs. 25/42 (59%)] or MSP1 [17/40 (42.5%) vs. 21/42 (50%)]. Dry season analysis revealed that whereas younger children had a slight decrease in the number of responders with detectable malaria antigen-specific MBC, a trend was noted towards a greater number of older responders: AMA1 [20/40 (50%) vs. 29/42 (69%); P = 0.08 b; χ^2^ analysis] or MSP1 [14/40 (35%) vs. 22/42 (52%); P = 0.11; χ^2^ analysis]. Linear regression models expressing malaria antigen-specific MBC as a function of age did not show significant associations. Of those tested, 29 individuals (35%) had no detectable MBC to either MSP1 or AMA1 at the wet season time point despite documented evidence of malaria infection in 28 individuals [(SP = 19 (36%); SN = 10 (34%)]. At the dry season follow-up period 6–9 months later, 12/29 [41% (or 15% of total population)] remained without detectable MBC to either malaria antigen [SP = 6 (32%); SN = 6 (60%); P = 0.13; χ^2^ analysis]. Of note, tetanus toxoid-specific MBC was examined in 20 children (aged 4–14 years) in an effort to incorporate a positive experimental control; however few positive responses were detected. This lack of tetanus toxoid response was not the result of technical issues since robust MBC were detected in a control U.S. malaria-naïve volunteer with known tetanus toxoid boosting 2 years prior to PBMC collection who was tested simultaneously (data not shown). The lack of detectable tetanus toxoid-specific MBC likely represents predictable loss of MBC more than two years following immunization [2], MBC below the level of detection, or lack of immunization. Tetanus toxoid was not used as a positive control in the remaining studies.

**Table 3:** Memory B cell (MBC) expressed as the mean number of *Schistosoma haematobium*-specific SFC to soluble egg antigen (SEA) or soluble worm antigen protein (SWAP) compared to total IgG SFC measured in children with (SP) or without (SN) *S. haematobium* infection and stratified by age group and season (i.e., malaria transmission and dry season).

Schistosoma Antigen	Age (years)	Group	MBC Transmission	MBC Dry	P value a
SWAP	4–14	SP	**0.017** b	**0.021** b	0.58
		SN	0.004	0.005	0.68
	4–8	SP	**0.019**	**0.019** b	0.94
		SN	0.003	0.003	0.98
	9–14	SP	**0.014** b	**0.022** b	**0.03**
		SN	0.007	0.006	0.62
SEA	4–14	SP	**0.034** b	**0.047** b	**0.05**
		SN	0.006	**0.012**	0.11
	4–8	SP	**0.024** b	**0.037** b	**0.04**
		SN	0.005	**0.015** c	0.18
	9–14	SP	**0.041** b	**0.055** b	0.26
		SN	0.005	0.009	0.44

a Statistical analysis performed between transmission and dry season values using the paired two-tailed ttest. P value deemed statistically significant ≤0.05.

b Statistical significance measured between SP and SN values using the Mann Whitney test for values not normally distributed (P value ≤0.05).

c Three of fifteen SN children had a positive response (ASC >0.01) to SEA. One child was subsequently determined to have acquired a *S. haematobium* infection in the interim since screening. All other responders to SWAP or SEA had repeatedly negative urine analyses for egg excretion.

ASC ratios of >0.01 were defined as positive specific MBC responses. Mean age-stratified group values >0.01 are depicted in bold font.

**Figure 2:**
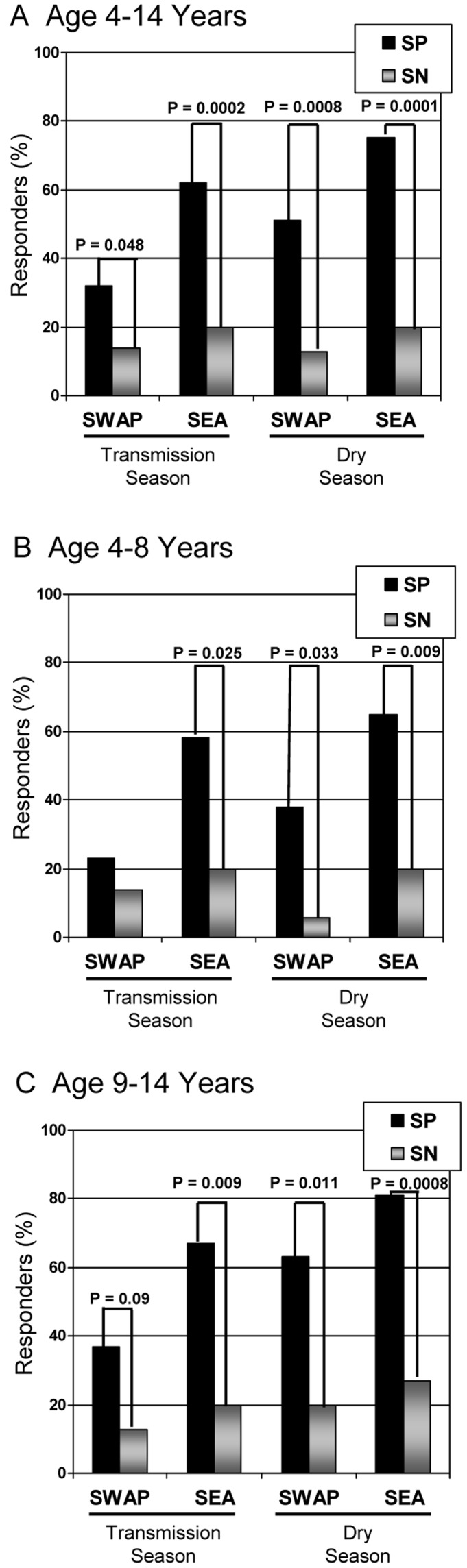
B memory cell responders to *S. haematobium* antigens. Shown are the percentages of B memory cell responders to *S. haematobium* antigens, SEA and SWAP, as determined by ELISpot assay during the malaria transmission season and again during the dry season with significant differences demarcated. Results were stratified by age (A–C) and *S. haematobium* status [*S. haematobium* positive (SP) = 54 and *S. haematobium* negative (SN) = 29] Responders are defined to those individuals with antigen specific cell (ASC) ratios (ratio of spot forming cells (SFC)/10^6^ expanded cells vs. total IgG SFC/10^6^ cells) greater than 0.01.

Children were next stratified by *S. haematobium* status and age group. In general, the mean ASC ratio of detectable MBC was increased in the transmission season as compared to the dry season and in the SP group compared to the SN group. However, this was only statistically significant in a few instances, notably between SP children aged 4–8 years and aged 4–14 years after stimulation with MSP1 when assessing the transmission season time point compared to the dry season results. This observation did not hold true for SP children aged 9–14 years. However, when analyzing the number of children deemed to have a positive ASC response (≥0.01), differences emerged upon age stratification. Whereas it appeared that little difference was noted in the ASC responses between SP and SN children when results were examined in children between ages 4–14 years, SP children aged 9–14 years of age exhibited significantly increased number of responders to both AMA1 and MSP1 in the transmission season than did the SN counterparts (AMA1∶20/27 (74%) vs. 5/15 (33%), P = 0.02; MSP1∶17/27 (64%) vs. 4/15 (27%), P = 0.05) using Fischer’s exact test (Figure 1C). Moreover, while the number of SP responders in the transmission season remained the same or fell slightly by the dry season follow-up, a notable increase in responders was noted in the SN group upon dry season follow-up (Figure 1C).

**Table 4:** Geometric mean antibody titers (OD 450 nm) stratified by antigen, season and schistosoma status in children with (SP) and without (SN) *S. haematobium*.

Cohort	Season	Antigen	Ab Titer c	SP vs. SNP Value^a^	Ab vs. MBC (ρ)^c^	95% CI^b^	P value	Ab vs. Age (ρ)^c^	95% CI^b^	P value
SP	Wet	AMA1	49,667	0.34	0.6567	0.5034–0.7699	**<0.0001**	0.1497	−0.0805–0.3648	0.19
SN			37,345							
SP	Dry		57,534	0.10	0.5229	0.3281–0.6750	**<0.0001**	0.1767	−0.0576–0.3926	0.13
SN			24,720							
Negative^d^			86							
Positive^d^			108,579							
SP	Wet	MSP1	34,172	0.56	0.4131	0.2034–0.5866	**0.0002**	0.2639	0.0398–0.4636	**0.02**
SN			50,081							
SP	Dry		25,956	0.51	0.4046	0.1920–0.5808	**0.0003**	0.3077	0.0847–0.5013	**0.006**
SN			20,568							
Negative			121							
Positive			58,292							
SP	Wet	SEA	25,539	**<0.0001**	0.5785	0.4016–0.7138	**<0.0001**	0.2910	0.0083–0.5306	**0.03**
SN			2,270							
SP	Dry		37,309	**<0.0001**	0.5355	0.3343–0.6751	**<0.0001**	0.1576	−0.1317–0.4221	0.27
SN			2,859							
Negative			473							
Positive			8,521							
SP	Wet	SWAP	4,511	**<0.0001**	0.3108	0.0866–0.5051	**0.006**	0.1690	−0.1201–0.4317	0.24
SN			818							
SP	Dry		7,238	**<0.0001**	0.2616	0.0316–0.4653	**0.02**	0.2856	0.0025–0.5264	**0.04**
SN			858							
Negative			126							
Positive			3,612							

a Statistical analysis performed between children with and without *S. haematobium* using the Mann Whitney test. P value deemed statistically significant ≤0.05. Statistical analysis between transmission and dry season values is not shown but no statistical significance was noted.

b 95% Confidence Interval of Spearman rank correlation test.

c Ab refers to geometric mean antibody and age refers to age-in-months. Spearman rank correlation (ρ) is depicted.

d Geometric mean antibody titers are reported for negative controls (10 U.S. malaria naïve adult sera) and positive controls (10 pooled Malian adult sera).

Also depicted are geometric mean negative and positive control values for each antigen. Spearman rank correlation (ρ) testing and 95% confidence interval depicting strength of association between antibody tested and MBC results as well as antibody tested and the child’s age in months.

**Figure 3:**
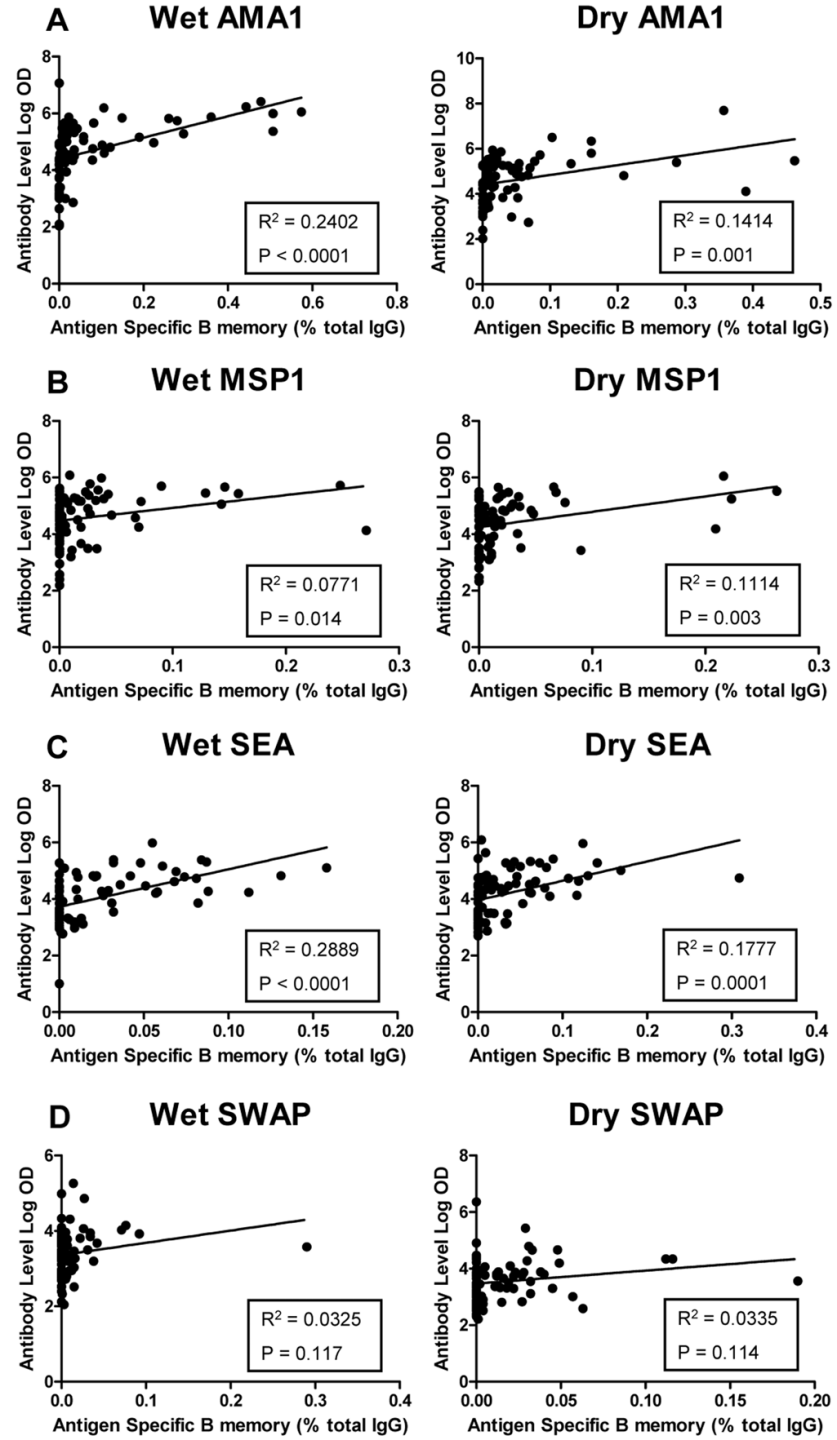
Correlation of antibody to B cell memory responses to *P. falciparum* and *S. haematobium* antigens. A–D show linear regression models demonstrating correlation between log-transformed antibody titers against *P. falciparum* antigens (AMA1 and MSP1) and *S. haematobium* antigens (SEA and SWAP) compared to antigen specific B memory during the malaria transmission (wet) and subsequent dry season. Each dot represents one individual. The solid line represents the best fit regression lines with R^2^ calculation and P value depicted.

### Memory B Cell Response to *S. haematobium* Antigens

Paired PBMC sample obtained at two time points were examined in SP and SN children (Table 3 and Figure 2). The ASC ratio was calculated after measuring *S. haematobium*-specific MBC to SEA and SWAP and expressed as a discreet value or as a positive (>0.01) response. The mean ASC ratio (Table 3) or total percentage of responders (Figure 2) stratified by time point, age group and *S. haematobium* status is depicted. Detectable MBC were significantly increased in SP children compared to SN children at both time points, in all age strata and to both antigens with the exception of children aged 4–8 years to SWAP (Figure 2). In general, increased detection of MBC in SP during the dry season SP MBC was noted in comparison to the transmission season and this phenomenon was noted to be statistically significant in younger children and in the group at large (aged 4–14 years) for SEA while older SP children had enhanced MBC to SWAP (Table 3). SEA appeared to elicit a more robust immune response with significantly more responders to SEA than to SWAP in both the transmission period and in the younger (4–8 years) age group. Of those children with known *S. haematobium* infection, 17/53 (32%) had no detectable MBC to either schistosoma antigen at the wet season time point. However, 11/17 (65%) developed demonstrable MBC response by the dry season follow-up. MBC to schistosoma antigens was detected in a small number of SN children. Repeated urine tests (n = 3) at screening were negative in all SN children. A total of 4 SN (13%) children had detectable MBC to SWAP and 7 SN (23%) children had MBC to SEA at the dry season follow-up. In no instance did individuals with detectable MBC to SWAP have detectable MBC to SEA (data not shown). Only one child was found to have acquired *S. haematobium* among the samples analyzed in this study, while the remainder had repeated negative urine tests for egg excretion at the dry season follow-up appointment. Correspondingly, this one individual had an eight-fold rise in antibody titer to SWAP (dry season titer = 16,777) and an end-point titer of 66,187 to SEA by the dry season follow-up.

### Flow Cytometric Analysis of Expanded Cells

We collected flow cytometric data on a subset of 14 participants (n = 7 SP and 7 SN children) to evaluate the composition of cells following *in vitro* expansion. We observed an increase in the percentage of CD19^+^CD3^−^ (total B cells) in all children evaluated. Pre-expansion total B cells were 13.8% (range 7.2–20.3%) of PBMC examined. Of these, 18.1% (range 11.1–33.4%) were Antibody-secreting plasmablasts/MBCs (CD19^+^CD27^+^CD38^+^CD3^−^). Post-expansion we observed a three-fold increase in the proportion of CD19^+^CD3^−^ (mean = 37.6%; range 26.6–51.8%), of which a mean of 51.5% (range 48.7–64.3%) were plasmablasts/MBCs (CD19^+^CD27^+^CD38^hi^CD3^−^) (Figure S1).

### Antibody Response to Malaria and *S. haematobium* Antigens

Paired sera sample obtained at two time points (wet season and dry season) were examined for the presence of IgG antibody to all four antigens (AMA1, MSP1, SEA and SWAP) (Table 4, Figure 3A–D). Serum was available from 84 children during the wet season and 82 during the dry season (2 sample did not have enough to sera complete ELISA to all antigens). The geometric mean (geomean) antibody titer for malaria antigens AMA1 and MSP1 did not vary by schistosoma status or by season. All children had measurable malaria antibody titers compared to U.S. malaria-naïve adults [AMA1 range 500–49,474,000; MSP1 range 250–1,222,076]. Malaria antibody titers were analyzed after stratifying by age-group. Values were consistently lower in the younger group (age 4–8 years) as compared to the older group (age 9–14 years), however this was only statistically significant for MSP1 antibody (Wet season geomean titer: 21,802 vs. 60,602, P = 0.02; Dry season geometric mean titer: 13,866 vs. 37,336, P = 0.017). Moreover, the significance was derived solely from SP children (Wet season geomean titer: 12,904 vs. 69,902, P = 0.005; Dry season geomean titer: 11,317 vs. 48,220, P = 0.009). No statistical significance was noted in age-stratified SN children. This finding also held true when evaluating the strength of the association between antibody and age (converted to months; Table 4).

Statistically significant higher antibody titers of SEA and SWAP were noted in SP children compared to SN children. Even children deemed negative for *S. haematobium* were found to have four-fold higher antibody titers than U.S. parasite-naïve adults (Table 4). A strong correlation, utilizing Spearman rank correlation coefficient, was noted between SEA antibody and the induction of MBC response, while a more modest correlation was noted between SWAP antibody and MBC response (Table 4). When the strength of association was examined between antibody response and age, a modest correlation was noted for SEA during the wet season and SWAP during the dry season. However, when we examined the strength of associations between antibody and urinary egg secretion, we found a stronger association (SEA – wet season: ρ = 0.3552, 95% CI [0.0768–0.5822], P = 0.01; dry season: ρ = 0.4462, 95% CI [0.1834–0.6494], P = 0.001) and (SWAP - wet season: ρ = 0.3014, 95% CI [0.0167–0.5409], P = 0.03; dry season: ρ = 0.4181, 95% CI [0.1498–0.6290], P = 0.003) suggesting that the burden of infection rather than the child’s age predicted antibody production. In turn, egg secretion did not correlate with MBC response to SWAP at any time point and only modestly correlated to SEA during the wet season (ρ = 0.2984, 95% CI [0.0251–0.5302], P = 0.03).

Linear regression models demonstrated a relatively modest correlation between log-transformed mean antibody titers to AMA1, MSP1 and SEA and antigen-specific MBC (Figure 3 A–C). This was not significant for SWAP (Figure 3D). However, a significant correlation was noted between the mean antibody titer and the MBC response by Spearman rank correlation coefficient for all 4 antigens tested (Table 4). In all cases the Spearman coefficient (ρ) was higher during the wet season than the dry. AMA1 appeared to have the strongest correlation during the wet season (ρ = 0.6567, P = <0.0001) while the dry season results for SWAP had the weakest correlation (ρ = 0.2616, P = 0.02) (Table 4).

In the face of markedly elevated antibody titers in SN children to both schistosoma antigens in comparison to U.S. parasite-naïve adults, we defined positive antibody responders for this endemic region as individuals with an antibody titer >3 SE above the SN mean (minus the top 10^th^ percentile of the SEA and SWAP antibody titers). Based on these criteria, positive antibody responses were titers of 2,880 and 1,825 for SEA and SWAP, respectively. Of SN children, 6/30 (20%) had detectable antibody titers to SEA and four of the six had detectable MBC. In regard to SWAP, 5/30 (17%) SN children had detectable levels, only one of whom had detectable MBC. In the SP cohort, 6/54 (11%) were negative for antibody to SEA, of whom only one had detectable MBC. In regard to SWAP, 11/54 (20%) were antibody negative, of whom 3 had detectable MBC. Thus, within the defined parameters, the sensitivity for SEA was determined to be 89% with a specificity of 80%. The sensitivity for SWAP was calculated to be 76% with a specificity of 83%.

## Discussion

There is growing evidence that immunologic B cell memory, albeit inefficient, develops to malaria blood-stage antigens [9]–[11], but very little is known regarding the acquisition of memory to other human parasitic infections or the immunologic perturbations that one parasitic infection has upon the immune responsiveness to another infection in the host. We demonstrate, for the first time, MBC response to *S. haematobium* antigens in Malian children with urinary egg excretion and provide evidence of seasonal acquisition of immunologic memory, age-associated differences in MBC acquisition, and correlation with circulating *S. haematobium* antibody. While durable MBC to malaria antigens in young children likely requires repeated infections with stepwise acquisition, we did not detect significant correlation of MBC to age over the 4–14 year old age distribution of Malian children. However, the presence of a parasitic co-infection resulted in older children with underlying *S. haematobium* infection having significantly more detectable MBC to malaria antigens (AMA1 and MSP1) than their age-matched SN counterparts.

Studies have consistently demonstrated inefficient generation of MBC across Africa and Southeast Asia to known blood-stage malaria antigens and vaccine candidates, AMA1 and MSP1, whether the analysis occurs in areas of high endemicity [9], seasonal endemicity [11], or low endemicity [10]. Similarly, we found comparable levels of antigen specific MBC in ∼60% of children to AMA1 and ∼44% to MSP1. While we demonstrate a trend towards a contraction of malaria antigen specific MBC seasonally and an expansion of the MBC compartment with age, this was only statistically significant for MSP1. Expansion of the malaria MBC compartment with age has been described; the greatest proportion of increases occur in individuals aged 18–25 years [11]; an age group which we did not examine. Stable antibody development to malaria antigens was detected and did not wane by the dry season follow-up. MSP1 antibody levels modestly correlated with age and were noted to be significantly higher in children aged 9–14 years, as compared to younger children aged 4–8 years. No such correlation was noted for AMA1. However, it is likely that a larger sample size might have detected smaller differences. Parasite antibody levels correlated modestly with MBC results, although we consistently noted that about one third of children with known parasitic infection (malaria or schistosomiasis), failed to mount any detectable MBC response. Over the course of seasonal follow-up, two thirds of these non-responders subsequently developed an MBC response. We speculate that this may be reflective of ongoing parasitic exposure and acquisition of memory. A longitudinal study with multiple time points over the course of several transmission seasons might be necessary to pinpoint the acquisition of memory to parasite antigens upon repeated infections. Alternatively, our observations may represent the immunosuppressive effect suggested in acute malaria [38]. It is also important to recognize that detectable MBC only reflect those in circulation at the time of specimen collection and those that respond to the strain of antigen used in stimulation, and may not represent the full sequestered MBC pool.

Immunologic MBC to *S. haematobium*, to our knowledge, has not been reported. Evidence of age-acquired immunity to schistosoma spp. does exist [39]. Studies examining the pathogenicity of *S. haematobium* found C3d, a breakdown product of complement C3, to be present in large quantities [40] and to correlate with egg production in *S. mansoni* models [16]. C3d contains the ligand for CD21, a B cell co-receptor, which would be expected to enhance B cell signaling when co-ligated to the B cell antigen receptors [41]. This might suggest that not only is the background milieu in schistosoma infections conducive to MBC development, but that non-specific bystander activation may induce enhanced response to other antigens. Our studies demonstrate robust MBC response in terms of both the numbers of responders and the magnitude of MBC responses to schistosoma antigens in children with known *S. haematobium* egg excretion as compared to SN children. Antibody levels correlated with MBC and with age, particularly for SEA. SEA is extremely immunogenic and generally elicited more detectable MBC (over 80% in children aged 9–14 years by the dry season time point) with higher ASC ratios (up to 0.055% of total IgG) than was measured to SWAP. Seasonal increases in the MBC compartment were noted to SEA in younger children aged 4–8 years and in the SP group in general. This was in contrast to SWAP where older children, aged 9–14 years, were noted to have seasonal acquisition (Table 3). It may be that SWAP is less immunogenic and stepwise acquisition of MBC occurs whereas a steady-state is achieved in older children in response to SEA.

Of note, 10–20% of SN children had evidence of MBC responses and/or detectable antibody titers to SEA or SWAP despite having repeated negative tests for egg excretion. There was no correlation between children who developed a MBC response to SEA compared to SWAP. However there appears to be an association between the minority of children who developed a MBC response to SEA and antibody elevation. One child was determined to have acquired *S. haematobium* in the interim from enrollment to dry season follow-up with corresponding increases in serum antibody levels. Peak acquisition of schistosomiasis occurs late in the dry season when standing water pools begin to dry, concentrating cercariae, which is unlikely to occur during malaria transmission. We cannot rule out the possibility that children may have had a previous *S. haematobium* infection and had been treated prior to study enrollment (however routine testing and treatment for individuals is uncommon until the regional population prevalence reaches 40% and all guardians denied previous infection in their children). Alternatively, children may have cleared a previous asymptomatic infection, or even, possibly, developed resistance to *S. haematobium* infection but remained free of active infection with egg excretion.

We have previously documented an age-associated protective effect against the acquisition of symptomatic malaria infection in children aged 4–8 years with concomitant *S. haematobium* [20]. Furthermore, we have shown immunologic perturbation in co-infected children including altered cytokine patterns, [25] and the presence of T regulatory cells [42]. In the case of MBC specific to malaria antigens, we noted a significant increase in detectable MBC to both MSP1 and AMA1 in the older group of children. As we have shown, an association exists between detectable MBC to SEA and to age and also to egg production. It is possible that older children have increased circulating C3d, which, in turn, stimulates MBC development to malaria antigens via non-specific activation of bystander B cells. Alternatively, the presence of *S. haematobium* may influence hyperresponsivity to TLR9 during an acute malaria infection. The immunosuppressive effect of parasitic infections upon TLR-mediated phenomena is well established [19], [43], [44]. Contradicting these findings, *S. mansoni-*infected mice have enhanced TLR9 responsiveness to SEA in bone marrow-derived Th2-primed macrophages [17]. Moreover, innate immune response to malaria infection is enhanced to TLR1, TLR2 and TLR4 agonists [45], as well as TLR9 [46]. Given that MBC stimulation can be driven by TLR4 and TLR9 [2], the presence of circulating *S. haematobium* moieties may potentiate MBC response to malaria antigens by a TLR pathway, primed to an acute malaria infection.

In conclusion, we have found detectable MBC to both malaria antigens and, for the first time, to schistosoma antigens and have shown evidence of enhanced immunologic memory responses to malaria antigens in children aged 9–14 years with *S. haematobium* infection. While this does not explain our clinical findings suggesting that *S. haematobium* confers an age-dependent protective effect in children aged 4–8 years against malaria, it does further emphasize the presence of strong immunologic interactions in co-parasitic infections. Future longitudinal studies are needed to further dissect the complexities of MBC development and to determine the role, if any, that this immunologic phenomenon plays in protection against malaria and the effect that TLR plays in mediating immunologic responses to parasitic infections.

## Supporting Information

Figure S1 **Representative flow cytometry plots depicting the gating strategy for detection of B cell memory responses.** Quantification of memory B cells (MBC) and MBC-derived antigen-secreting cells (ASC, plasmablasts) with representative plots depicting the gating strategy. The percentages of gated populations are denoted in the gated areas of each histogram. Cells that were determined to be Vivid^-^CD14^-^CD3^-^CD19^+^ (Fig. A–C) were then examined for the presence of CD38 and CD27. Doublets and aggregates were gated out (histogram not shown). Results pre- (Fig. D) and post- (Fig. E) PBMC expansion with mitogens (pokeweed mitogen, β-mercaptoethanol, CpG-2006 and *Staphylococcus aureus* Cowan) are depicted. CD27^+^CD38^+^ cells can be divided into those determined to be CD38^hi^ (i.e., ASC/plasmablasts depicted in the top half of the gates in panels D and E) and CD38^dim^ (i.e., memory B cells depicted in bottom half of gates in panels D and E). (Note: Data is reported as a combined population to illustrate total in vitro expansion).
(TIF)

Table S1 **Memory B cell (MBC) expressed as the mean number of** ***P. falciparum*** **or** ***S. haematobium*** **antigen specific MBC to apical membrane antigen (AMA1), merozoite surface protein 1 (MSP1), soluble worm antigen protein (SWAP), or soluble egg antigen (SEA) compared to total IgG ASC (i.e., ASC ratio) measured in children with** ***S. haematobium*** **(SP) infection with malaria (SP Mal) or without malaria (SP no Mal) infection.** Results are stratified by age group and season (i.e., malaria transmission and dry season). ASC ratios ≥0.01 were defined as a positive specific MBC responses. Mean age-stratified group values ≥0.01 are depicted in bold font.
(DOC)
