# Supplementary figures and images for: Antigen-Specific B Memory Cell Responses to Plasmodium falciparum Malaria Antigens and Schistosoma haematobium Antigens in Co-Infected Malian Children
Source: PLoS One. 2012 Jun 5;7(6):e37868. doi: 10.1371/journal.pone.0037868 (PMC3367916; doi:10.1371/journal.pone.0037868)

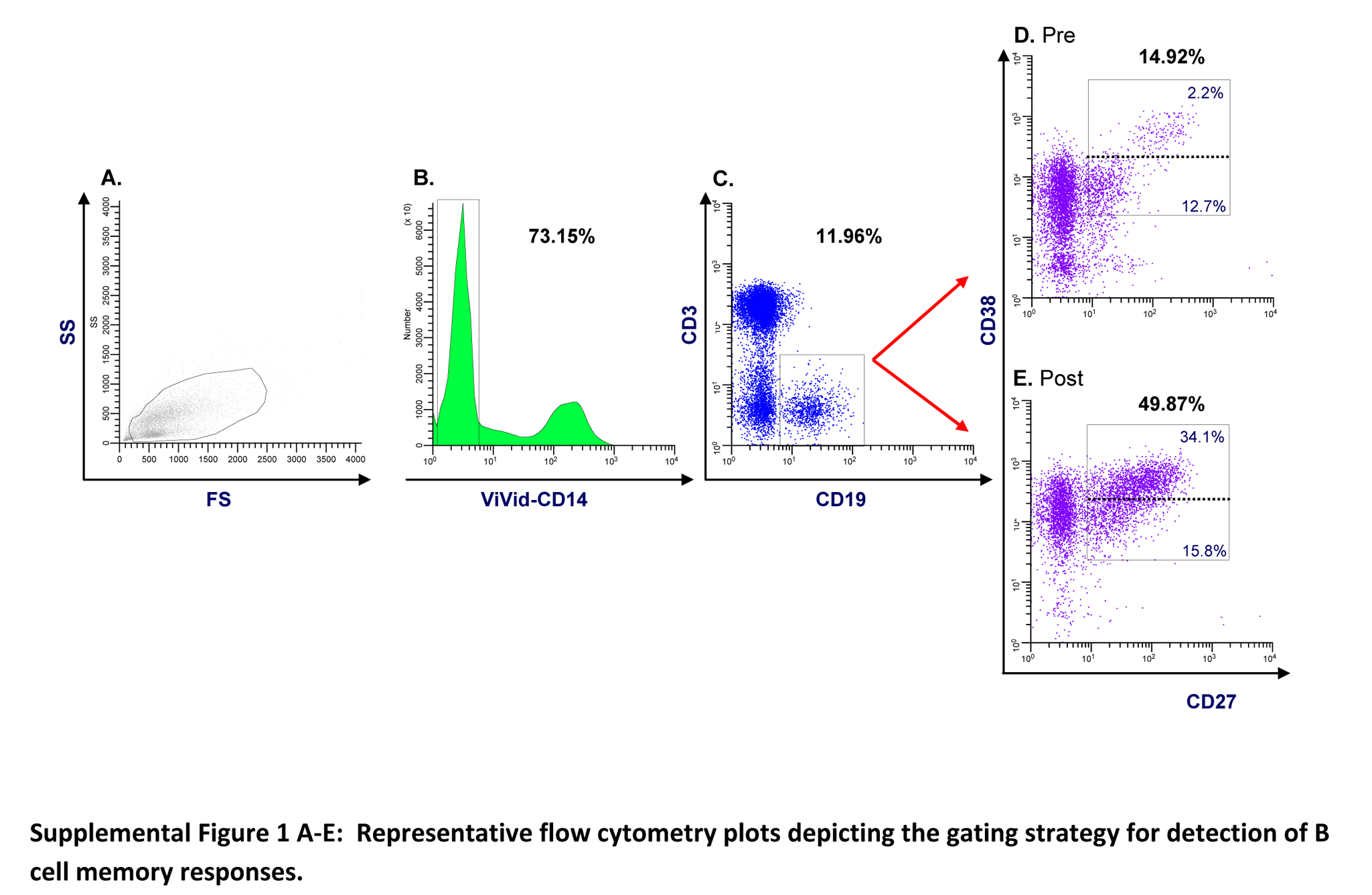

Supplement: Figure S1 — Representative flow cytometry plots depicting the gating strategy for detection of B cell memory responses. Quantification of memory B cells (MBC) and MBC-derived antigen-secreting cells (ASC, plasmablasts) with representative plots depicting the gating strategy. The percentages of gated populations are denoted in the gated areas of each histogram. Cells that were determined to be Vivid-CD14-CD3-CD19+ (Fig. A–C) were then examined for the presence of CD38 and CD27. Doublets and aggregates were gated out (histogram not shown). Results pre- (Fig. D) and post- (Fig. E) PBMC expansion with mitogens (pokeweed mitogen, β-mercaptoethanol, CpG-2006 and Staphylococcus aureus Cowan) are depicted. CD27+CD38+ cells can be divided into those determined to be CD38hi (i.e., ASC/plasmablasts depicted in the top half of the gates in panels D and E) and CD38dim (i.e., memory B cells depicted in bottom half of gates in panels D and E). (Note: Data is reported as a combined population to illustrate total in vitro expansion). (TIF) [file pone.0037868.s001.tif]
