# Supplementary material for: Antigen-Specific B Memory Cell Responses to Plasmodium falciparum Malaria Antigens and Schistosoma haematobium Antigens in Co-Infected Malian Children
Source: PLoS One. 2012 Jun 5;7(6):e37868. doi: 10.1371/journal.pone.0037868 (PMC3367916; doi:10.1371/journal.pone.0037868)
Supplement: Table S1 — Memory B cell (MBC) expressed as the mean number of P. falciparum or S. haematobium antigen specific MBC to apical membrane antigen (AMA1), merozoite surface protein 1 (MSP1), soluble worm antigen protein (SWAP), or soluble egg antigen (SEA) compared to total IgG ASC (i.e., ASC ratio) measured in children with S. haematobium (SP) infection with malaria (SP Mal) or without malaria (SP no Mal) infection. Results are stratified by age group and season (i.e., malaria transmission and dry season). ASC ratios ≥0.01 were defined as a positive specific MBC responses. Mean age-stratified group values ≥0.01 are depicted in bold font. (DOC) [file pone.0037868.s002.doc]

**Table S1:** Memory B cell (MBC) expressed as the mean number of *P. falciparum* or *S. haematobium* antigen specific MBC to apical membrane antigen (AMA1), merozoite surface protein 1 (MSP1), soluble worm antigen protein (SWAP), or soluble egg antigen (SEA) compared to total IgG ASC (i.e., ASC ratio) measured in children with *S. haematobium* (SP) infection with malaria (SP Mal) or without malaria (SP no Mal) infection. Results are stratified by age group and season (i.e., malaria transmission and dry season). ASC ratios >0.01 were defined as a positive specific MBC responses. Mean age-stratified group values >0.01 are depicted in bold font.

| **Antigen** | **Age (years) ^a^** | **Season** | **MBC SP no Mal** | **MBC SP Mal** | **P value ^b^** |
| --- | --- | --- | --- | --- | --- |
| AMA1 | 4-14 | Transmission | **0.098** | **0.080** | 0.65 |
|  |  | Dry | **0.075** | **0.065** | 0.63 |
|  | 4-8 | Transmission | **0.059** | **0.079** | 0.66 |
|  |  | Dry | **0.044** | **0.045** | 0.92 |
|  | 9-14 | Transmission | **0.151** | **0.076** | 0.26 |
|  |  | Dry | **0.127** | **0.074** | 0.32 |
| MSP1 | 4-14 | Transmission | **0.034** | **0.033** | 0.75 |
|  |  | Dry | **0.019** | **0.019** | 0.99 |
|  | 4-8 | Transmission | **0.016** | **0.024** | 0.72 |
|  |  | Dry | 0.006 | 0.008 | 0.74 |
|  | 9-14 | Transmission | **0.043** | **0.033** | 0.80 |
|  |  | Dry | **0.032** | **0.029** | 0.91 |
| SWAP | 4-14 | Transmission | **0.01** | **0.019** | 0.47 |
|  |  | Dry | **0.017** | **0.021** | 0.68 |
|  | 4-8 | Transmission | 0.005 | **0.023** | 0.47 |
|  |  | Dry | 0.007 | **0.019** | 0.48 |
|  | 9-14 | Transmission | **0.015** | **0.013** | 0.88 |
|  |  | Dry | **0.029** | **0.022** | 0.60 |
| SEA | 4-14 | Transmission | **0.027** | **0.035** | 0.50 |
|  |  | Dry | **0.032** | **0.047** | 0.37 |
|  | 4-8 | Transmission | **0.029** | **0.019** | 0.49 |
|  |  | Dry | **0.036** | **0.037** | 0.96 |
|  | 9-14 | Transmission | **0.024** | **0.041** | 0.21 |
|  |  | Dry | **0.024** | **0.05** | 0.20 |

**^a^** A total of 39 SP Mal (26 aged 4-8 years, 27 aged 9-14 years) and 14 SP no Mal (7 aged 4-8 years, 7 aged 9-14 years) were examined in both the transmission and dry season (Note: Sample for 1 SP Mal from the wet and the dry season was excluded).

**^b^** Statistical significance measured between SP and SN values using the Mann Whitney test for values not normally distributed (P value < 0.05).
